# Closed trans-intersphincteric fistulotomy: a new modified sphincter-sparing technique for high transsphincteric anal fistula

**DOI:** 10.3389/fsurg.2024.1487245

**Published:** 2024-12-18

**Authors:** Bo Chen, Yueting Wang, Zubing Mei, Chang Mao, Yicheng Liu, Wenjun Zhao, Yingying Li, Qianqian Ye, Jin Xu, Qingming Wang

**Affiliations:** ^1^ Department of Anorectal Diseases, Shanghai Baoshan District Integrated Traditional Chinese and Western Medicine Hospital, Shanghai, China; ^2^ Department of Traditional Chinese Medicine, Juquan Xincheng Community Health Service Center, Shanghai, China; ^3^ Department of Anorectal Surgery, Shuguang Hospital Affiliated to Shanghai University of Traditional Chinese Medicine, Shanghai, China

**Keywords:** closed trans-intersphincteric fistulotomy, sphincter-sparing technique, high transsphincteric anal fistula, clinical efficacy, fistula-in-ano

## Abstract

**Background:**

The main goals of surgery for fistula-in-ano are to completely resolve the condition and maintain optimal anal function. Effective management of the internal opening during and proper postoperative drainage of the intersphincter plane are crucial for achieving successful outcomes. This study evaluated the clinical efficacy of a novel sphincter-sparing technique for treating high transsphincteric anal fistula (HTAF).

**Methods:**

This prospective study included 55 patients with HTAF who underwent closed trans-intersphincteric fistulotomy (CTiF) between July 2021 and April 2022 at our institution. Preoperative anorectal magnetic resonance imaging was performed for all patients. The primary outcome measures assessed the rate of fistula healing while the secondary outcome measures evaluated healing time, Cleveland Clinic Florida fecal incontinence score (CCF-FIS), 11-point visual analog scale (VAS) pain score and postoperative complications.

**Results:**

We included 55 patients with HTAF in this study. During a mean follow-up period of 9.3 months, CTiF achieved a healing rate of 90.91% (50/55). The mean time to recovery was 7.09 ± 1.94 weeks. Four (7.27%) patients developed postoperative urinary retention. At the 6-month follow-up, the CCF-FIS and VAS score were 0 [(0,0) range, 0–3] and 0 [(0,1); range, 0–4], respectively. Two patients with recurrent HTAF recovered after treatment with a transanal opening of intersphincteric space procedure, and three recovered after seton placement.

**Conclusions:**

CTiF is a promising and effective sphincter-sparing technique for treating HTAF. To confirm long-term outcomes, larger sample size prospective randomized controlled trials are required.

## Introduction

The management of high complex anal fistulas presents a formidable challenge to colorectal surgeons worldwide (1). This condition, characterized by its complex anatomical pathways, has the potential to result in a high rate of recurrence, as well as incontinence and other significant complications. High transsphincteric anal fistula (HTAF) is a complex anal fistula that accounts for 30% of all anal fistulas; it passes through the internal anal sphincter (IAS) and involves more than one-third of the external anal sphincter (EAS) (2, 3). The deep intersphincteric space (DPIS) is a crucial anatomic site in the propagation of complex cryptoglandular anal fistulas, which play an important role in the formation of HTAF (4, 5). In particular, the primary infection originating from the anal glands tends to propagate toward the DPIS. Subsequently, HTAF is formed when the sepsis traverses the IAS and EAS and reaches ischiorectal fossa (6, 7). Precise knowledge of the exact position of the anal fistula and its relation with the anatomical structures is essential for the successful treatment of HTAF.

Fistulotomy is universally accepted and widely used to treat simple anal fistulas. However, the aggressive laying-open technique may cause unavoidable harm to the anal sphincter, leading to different levels of postoperative incontinence (8). With the development of surgical techniques, the treatment of anal fistulas has ranged from straightforward standardized incision and drainage to very complex sphincter-sparing procedures. These innovative techniques include ligation of the intersphincteric fistula tract (LIFT) (9); endorectal advancement flap (10); video-assisted anal fistula treatment (11); and anal fistula plug (12), and the injection of autologous centrifuged adipose tissue (13). Despite these advances, their long-term performance often fails to meet expectations. Closed trans-intersphincteric fistulotomy (CTIF) is an innovative surgical technique developed from LIFT (9), representing a progression for treating of HTAF. This technique, characterized by its conservative approach to the the anal sphincter complex, involves excising the intersphincteric fistula and associated infection via an intersphincteric approach while also addressing the primary internal opening. Subsequently, the IAS is meticulously sutured in a continuous manner to maintain its barrier function and minimize the likelihood of the constant presence of stool at the surgical site. Secondary healing is accomplished through the implementation of proper drainage of the intersphincteric incision. This study evaluated the safety and efficacy of CTiF technique in treating HTAF through a comprehensive analysis of clinical outcomes.

## Materials and methods

### Study design

This was a prospective study conducted on patients treated with the CTiF technique between July 2023 and April 2024 in a large tertiary medical center in Shanghai (Shuguang Hospital Affiliated with Shanghai University of Traditional Chinese Medicine). This study was approved by the ethics committee of Shuguang Hospital Affiliated with Shanghai University of Traditional Chinese Medicine (Study Approval No. 2023-1270-27-01). Written consent was obtained from all patients who were informed about the procedure. Inclusion criteria were patients aged ≥18 years presenting with HTAF. HTAF was diagnosed in accordance with the Parks classification (2), specifically identifying fistulas with tracts crossing >30%–50% of the external sphincter, including primary and recurrent cases involving multiple tracts. The diagnosis of cryptoglandular fistula was confirmed through magnetic resonance imaging (MRI) and intraoperative examination under anesthesia in all patients. Exclusion criteria were patients with inflammatory bowel disease, HIV infection, previous surgery for a malignant neoplasm during past five years, previous pelvic radiotherapy, autoimmune diseases, pregnant or breastfeeding women, and patients unable to provide informed consent. Rectovaginal fistulas were excluded.

### Procedure

#### Preoperative preparation

**Figure 1:**
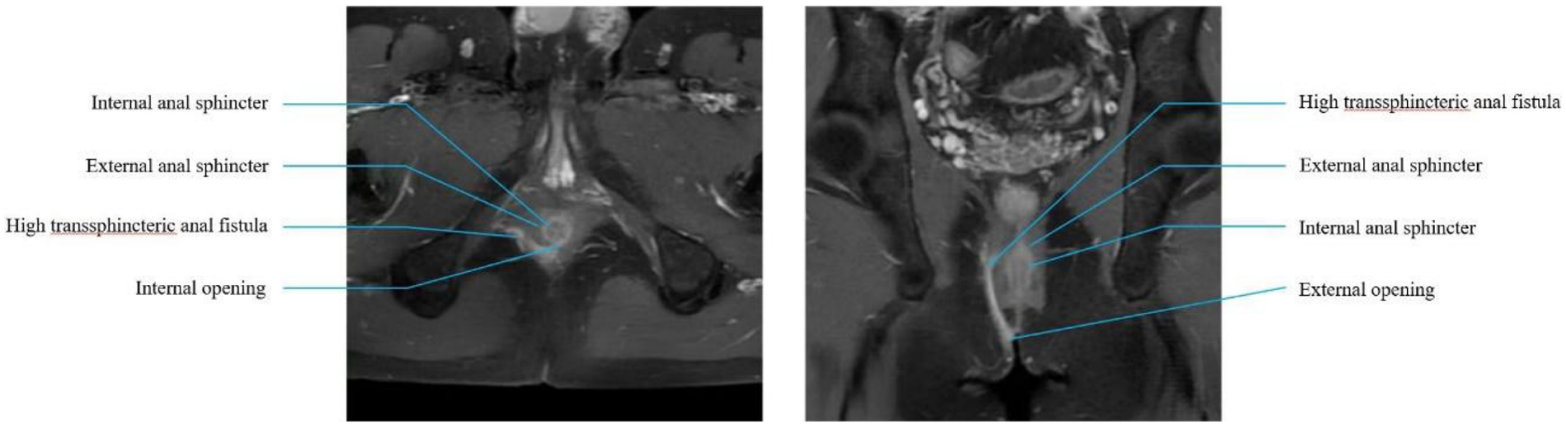
Preoperative MRI. Lithotomy and coronal section of preoperative MRI scan.

All patients underwent MRI before surgery to delineate the HTAF tracts and their anatomic relationship with the sphincter. This imaging was used to facilitate accurate identification of the internal opening and tracts, thereby providing direction for surgical planning (Figure 1). Patients received a glycerin enema 1 h before the procedure. Surgery was performed with the patient in the lithotomy position under spinal anesthesia.

**Figure 2:**
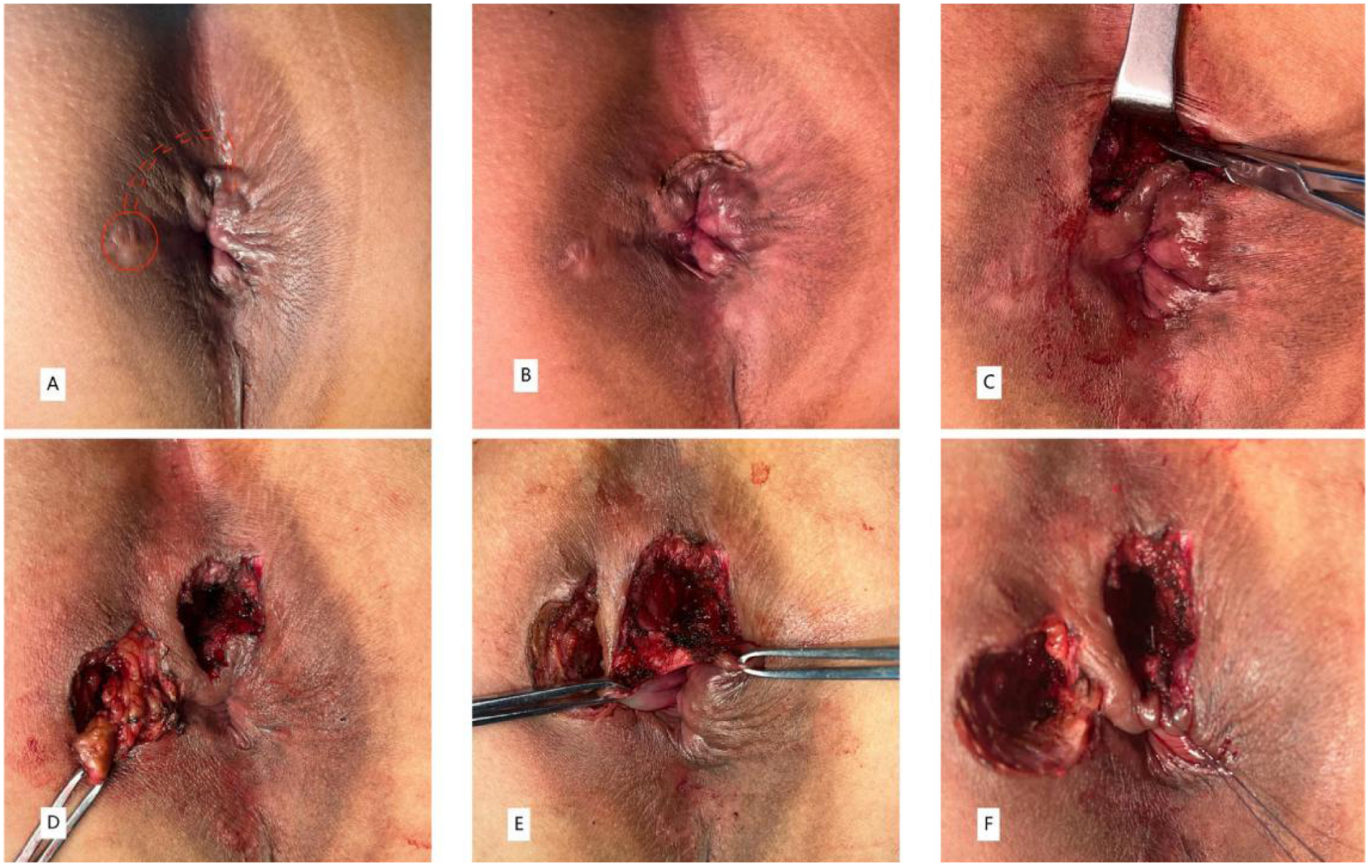
A 35-year-old man with HTAF treated by the CTiF procedure with complete healing demonstrated on follow-up **(A–F)**.

(1) A curvilinear skin incision was made at the site of the fistulous tract between internal anal sphincter (IAS) and external anal sphincter (EAS) to enter the intersphincteric groove (IS approach) (Figure 2B). With meticulous dissection of the intersphincteric space by an electrical scalpel, the intersphincteric tracts were identified and isolated (Figure 2C). (2) Careful incision and electrical cautery was performed to obliterate the intersphincter tract and sepsis without injuring the sphincter. Thereafter, the intersphincteric space was irrigated repeatedly with povidone iodine and hydrogen peroxide and povidone iodine to clear the sepsis. (3)The fistula from the external orifice to the EAS was dissected in a tunnel-based way, and the defect on the EAS was sutured with a 2-0 polyglactin purse string suture (Figure 2D). The location of the internal orifice was identified through the intersphincteric fistula. (4) Once determined, a small curved clamp was inserted through the internal orifice located within the intersphincteric space into the anal canal. Subsequently, performed an incision on the IAS extending from the internal opening to the anal verge (Figure 2E). Following this, employed absorbable sutures to continuously and securely suture the IAS through the intersphincteric space. Lastly, the intersphincteric plane was kept open and iodophor cotton slivers were placed for drainage (Figure 2F).

#### Postoperative management and follow-up

Postoperatively, all patients were prescribed 2.0 g intravenous cefmetazon, twice a day (1.0 g sildenafil was given intravenously once a day for penicillin-allergic patients). The duration of intravenous anti-inflammatory drugs was determined according to the severity of the anal fistula and the length of the hospital stay. Iodophor cotton slivers were used to clean the wound and were placed at the intersphincteric plane for drainage 2 times a day until the wound healed. Patients were considered to have postoperative recurrence if they experienced the following conditions:(1) previous wounds developed erythema, swelling, fluctuation, pus discharge from external openings; (2) self-reported pain and/or pain during palpation; (3) previous wound that did not heal in 3 months after surgery (14).

All patients were examined at the outpatient clinic by the CTIF surgeons once a week for 6 weeks. Thereafter, telephone follow-up was conducted every 4 weeks. Patient demographics, clinical information, and short-term clinical outcome data were noted through outpatient and telephone follow-up.

### Study outcomes

The primary outcome measure of this study was the 6-month healing rate. The standard for complete healing of HTAF is when the skin of all fistula tract areas is free from tenderness, without discharge of pus from any canal or the anus (15). Secondary outcome measures included Cleveland Clinic Florida fecal incontinence score (CCF-FIS) (16), the 11–point visual analog scale (VAS) pain score, and postoperative complications (17). The VAS pain score was evaluated and used to assess patients' postoperative pain: 0 indicates no pain, 1–3 indicates slight pain, 4–6 indicates pain affecting sleep, and 7–10 indicates severe intolerable pain and inability to sleep. The CCF-FIS, which includes stool morphology, gastrointestinal gas incontinence, wearing pads, and lifestyle, with a score range of 0–20, was used to assess the severity of fecal incontinence symptoms.

### Statistical analysis

SPSS Statistics 25.0 (IBM Inc., IL, USA) software was employed for statistical analysis. Data for continuous variables were reported as mean ± standard deviation (SD) or median with interquartile range (minimum—maximum). Continuous variables were compared using the independent *t*-test for normal distribution and the Mann–Whitney *U* test for non-normal distribution. A *P* value < 0.05 was considered statistically significant.

## Results

**Figure 3:**
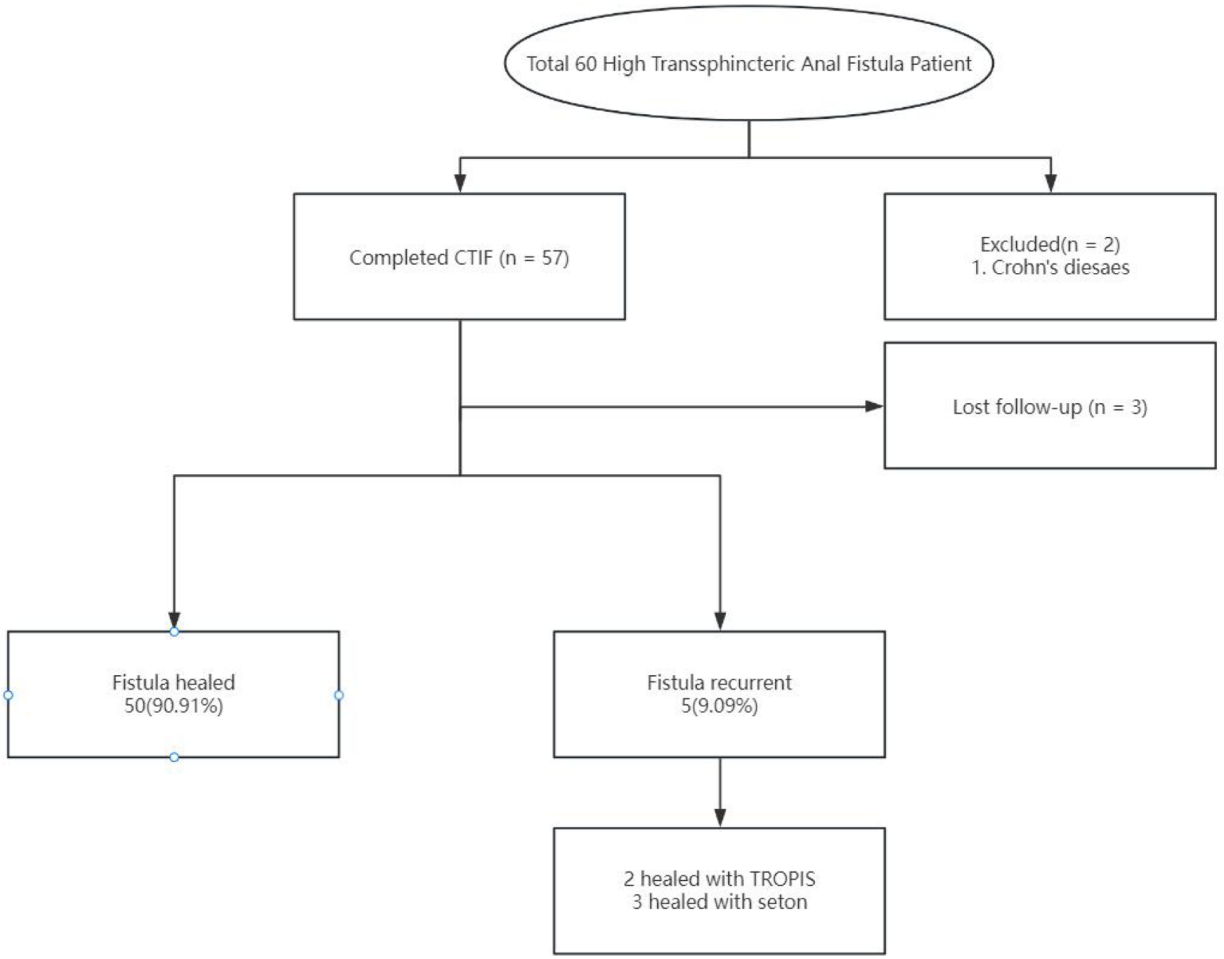
Flow chart of the short-term outcome in a patient treated with CTiF.

Total 60 patients with HTAF were prospectively enrolled and treated with CTIF. Three patients were lost to follow-up before the 6-month visit, and 2 patients with an anal fistula associated with Crohn's disease were excluded; thus, 55 patients were ultimately included in this study (Figure 3). The ratio of male/female was 51/4; the mean age was 36.04 ± 9.80 years; the mean body mass index was 24.34 ± 3.12 kg/m^2^; 3 (5.45%) patients had diabetes; and 7 (12.73%) patients had hypertension. Patient characteristics are shown in Table 1. The mean surgical duration was 28.6 ± 4.58 min. Four (7.27%) patients showed postoperative urinary retention: one was administered an intramuscular neostigmine injection, and the other three were catheterized. Details of the surgery are shown in Table 2.

**Table 1:** Demographic and baseline characteristics for patients.

Parameter	Patients (*N* = 55)
Sex, *n* (%)	
Female	4 (7.27)
Male	51 (92.73)
Age, (mean ± SD)	36.04 ± 9.80
Age range (min-max)	18–59
Height, m(mean ± SD)	1.74 ± 0.06
Weight, kg(mean ± SD)	73.84 ± 10.52
BMI, kg/m^2^ (mean ± SD)	24.34 ± 3.12
Diabetes mellitus, *n* (%)	3 (5.45)
Hypertension, *n* (%)	7 (12.73)
Recurrence history, *n* (%)	9 (16.36)
Previous perianal procedure (any), *n* (%)	16 (29.09)
History of abscess drainage, *n* (%)	10 (18.19)

Data presented as median (IQR) or number (%) BMI, body mass index.

**Table 2:** Operation data and clinical outcomes.

Parameter	Patients (*N* = 55)
Overall healing rate, *n*	50 (90.91)
Duration of surgery, min (mean ± SD)	28.6 ± 4.58
Intraopeative blood loss, ml (mean ± SD)	22.91 ± 5.83
Time to recovery (weeks)	7.09 ± 1.94
Complication, *n* (%)	
Bleeding	0 (0)
Fecal/gas incontinence	0 (0)
Urinary retention	4 (7.27)
Other	0 (0)
Time to return to work/activities, days [M(P25, P75), d]	5 (3,7)
Follow-up period, months (mean ± SD)	9.2 ± 1.87

At the 6-month follow-up, the CCF-FIS and VAS score were 0 [(0,0) range, 0–3] and 0 [(0,1); range, 0–4], respectively. The main results are shown in Table 3. During a mean follow-up period of 9.3 months (SD 1.81; range, 6–15), 50 (90.91%) patients recovered after the CTIF procedure, and there was recurrence of symptoms in 5 (10.91%) patients. The mean time to recovery was 7.09 ± 1.94 weeks. Among these 5 recurrent fistulas, 2 had impaired healing of the internal opening at 5 weeks and 6 weeks after surgery, and 3 patients had sepsis in the intersphincteric plane due to poor drainage at 4 weeks, 5 weeks, and 7 weeks. Two recurrent patients recovered after treatment with a TROPIS procedure, and three recovered after seton placement. No patient had anal incontinence.

**Table 3:** Perioperative and postoperative patient data.

Parameter	PRD-OP1	POD30	POD60	POD180
CCF-FIS [M(P25, P75)]	3 (1.5)	2 (1.3)	1 (0.2)	0 (0.0)
VAS score [M(P25, P75)]	3 (1.5)	2 (1.3)	1 (0.2)	0 (0.1)

PRD, preoperative day; POD-OP, postoperative day; CCF-FIS, Cleveland Florida Clinic incontinence score; VAS score, visual analog scale pain score.

## Discussion

HTAF tracts are complex. Their formation is closely related to the unique anatomical factors that make them prone to infection and spreading. Zhang et al. (5) conducted a retrospective MRI analysis of 508 patients with anal fistula and found that DPIS is often involved in complex posterior cryptoglandular fistulas and is involved in 80.1% of cases with HTAF. Chronic inadequate drainage of sepsis within DPIS is a principal factor contributing to recurrent postoperative episodes (18, 19). Therefore, DPIS is crucial during and after surgery in the management of HTAF.

Conventional surgical interventions, which involve extensive resection of the anal fistula, inflict considerable damage on the anal sphincter, precipitating incontinence and protracted recovery. Although recent advances in sphincter-preserving methodologies have been recognized for their safety, the results of these procedures are variable and fail to show the superiority of one procedure over another (20). The persistent risk of potential damage to the anal sphincters, along with the subsequent adverse functional outcomes, remains significant in patients with HTAF. Currently, transsphincter anal fistulas are primarily treated with the LIFT procedure. Nevertheless, this surgical approach is predominantly advisable for transsphincter anal fistulas without another branch, and it carries a high risk of long-term recurrence. Sun et al. (21) estimated that the LIFT procedure cure rate for treating HTAF ranges 25%–92%. In contrast to the ligation of fistulas by the LIFT procedure, tunnel resection was performed on fibrotic fistula tracts from the intersphincteric plane to the external opening in our surgery. Moreover, high anal fistula requires continuous adequate drainage of pus after surgery until the cavity is completely healed; therefore, we expected that opening the intersphincter plane would have a more beneficial effect.

In 2017, Garg et al. (22) focused on the treatment of the sphincter space and proposed a new sphincter-preserving operation: the TROPIS procedure. The fistula and internal opening were treated by direct incision of the mucosa from the internal opening to outside the IAS, deroofing the intersphincteric space from the luminal side. In a long-term follow-up study, however, it was found that the one-time cure rate of the TROPIS procedure in the treatment of high complex anal fistula was only 78.4% (23). Upon investigation, the author believes that the recurrence may be related to the failure to accurately locate the internal opening and thoroughly remove the intersphincteric fistulous tract. Mei et al. (17) found that an unidentified internal opening significantly correlated with the recurrence of anal fistula after surgery (relative risk, 8.54).

CTiF divide the surgical site into two parts: the IAS part and the EAS part, based on the intersphincteric space. In the intersphincteric space, the internal opening can be found more accurately along the direction of fistula, so as to reduce the risk of postoperative recurrence. Although we cut a small portion of the IAS during the operation (similar to the TROPIS procedure), we preserved the EAS and performed reconstruction and suturing of the IAS. The impact on anal function is minimal, and the study results have confirmed this. In addition, the IAS should be sutured to maintain the barrier effect of the IAS. Continuous suture with absorbable suture is a common choice, because absorbable suture can reduce the discomfort and complications of postoperative suture removal. When suturing, it is necessary to ensure the tightness and tension of suture is moderate to avoid postoperative complications such as fistula recurrence or anal stenosis. Within the scope of this study, we explored CTiF as a novel, sphincter-conserving stratagem, demonstrating its viability and safety. Remarkably, this technique achieved a healing rate of 90.91% (50 of 55 cases), with a median recuperation timeframe of 7.09 weeks during the mean follow-up period of 9.3 months. The outcomes were superior relative to existing sphincter-sparing approaches. Furthermore, given that anal fistulas may arise from diverse factors, such as anastomotic fistulas caused by colorectal cancer surgery and rectovaginal fistulas resulting from perineal colostomies, it is crucial to closely monitor this complication (24). Butyrylcholinesterase, serving as a new predictive biomarker of postoperative complications following colorectal surgery, can be considered to detect the recurrence of anal fistulas (25). The CTiF treatment points are discussed below.

### Necessity of preoperative MRI

This imaging modality is instrumental in delineating fistula tracts, notwithstanding the incremental cost implications. Its precision in identifying the internal opening and sepsis facilitates the preservation of anal function and anatomical integrity by mitigating the risk of inadvertent damage during the disentanglement of IAS and EAS. Preservation of these muscular structures is of paramount importance because IAS is a longitudinal muscle bundle and EAS is a transverse muscle bundle. A cohort study by Buchanan et al. (26) employing MRI to investigate the trajectory of the fistula tract in transsphincteric anal fistulas demonstrated that the fistula can form through the EAS at any angle, and the angle of the transsphincteric fistula passing through the sphincter complex is clearly shown on MRI axial or coronal images. They also found that some fistulas would cross the EAS obliquely upward, resulting in the fistula and internal opening being on different levels. This type of anal fistula often causes greater damage to the anal sphincter during surgery and is prone to cause anal incontinence. Therefore, accurate preoperative imaging techniques are instructive for the treatment of anal fistulas and for maintaining anal function.

### Postoperative care protocols mandate rigorous management of the anal and intersphincteric planes

Three patients had intersphincteric sepsis due to poor drainage at 4, 5, and 7 weeks. Thus, it was necessary to use iodophor cotton slivers to clean the wound and place them at the intersphincteric groove for drainage 2 times a day (27).

This study has several limitations. As a single-center investigation with a limited cohort, the potential for selection bias during the recruitment phase cannot be overlooked. Additionally, the absence of postoperative MRI or endoanal ultrasound evaluations in our protocol could lead to the underdiagnosis of recurrent conditions.

## Conclusion

CTIF is a safe and effective sphincter-sparing technique for managing HTAF and is worth clinical promotion. Further multicenter, randomized controlled trials comparing CTlF with other techniques and involving larger sample sizes are required to confirm the effectiveness and safety of this procedure.

## Data Availability

The original contributions presented in the study are included in the article/Supplementary Material, further inquiries can be directed to the corresponding authors.
